# Book Reviews

**Published:** 1901-11

**Authors:** 


### BOOK REVIEWS.

The Pathology and Treatment of Sexual Impotence. By Victor G. Vecki, M.D. Third Edition, Revised and Enlarged. 12mo, 329 pages. Philadelphia and London: W. B. Saunders & Company, 1901. Cloth, $2.00 net.

The reading part of the medical profession of America and England has passed judgment on this monograph. The whole subject of sexual impotence and its treatment is discussed by the author in an exhaustive and thoroughly scientific manner. The former edition was exhausted in less than two years. In this edition the book has been thoroughly revised, and new matter has been added, especially to the portion dealing with treatment.

Although no one denies that the sexual function is of the very greatest consequence to the individual as well as to society in general, yet the subject of impotence has but seldom been treated in this country in the truly scientific spirit that its pre-eminent importance deserves, and this volume will come to many as a revelation of the possibilities of therapeutics in this important field. The Author ventures to assert that in many cases it is a better deed to restore to an impotent man the power so precious to every individual, than to preserve a dangerously sick person from death, for in many cases death is preferable to impotence.

It is a well-written, scientific work, and can be recemmended as a scholarly treatise on its subject.

---

The Principles of Hygiene: A Practical Manuel for Students, Physicians and Health Officers. By D. H. Bergey, A.M., M.D., First Assistant, Laboratory of Hygiene, University of Pennsylvania. Octavo volume of 495 pages, illustrated. Philadelphia and London: W. B. Saunders & Company, 1901. Cloth, $3.00 net.

This book is intended to meet the needs of students of medicine in the acquirement of a knowledge of those principles upon which modern hygienic practices are based, and to aid physicians and health officers in familiarizing themselves with the advances made in hygiene and sanitation in recent years. The book is based on the most recent discoveries, and represents the practical advances made in the science of hygiene up to date.

Among the important subjects considered are Ventilation, Heating, Water and Water Supplies, Disposal of Sewage and Garbage, Food and Diet, Exercise, Clothing, Personal Hygiene, Industrial Hygiene, School Hygiene, Military and Naval Hygiene, Habitations, Vital Statistics, Disinfection, Quarantine, etc. The idea of the book is to give the reader a clear understanding of the general principles of this broad subject. The rapid strides made in our knowledge of the entire subject has rendered such a book, reflecting the more recent discoveries, a necessity to physicians and students of medicine.

---

PROGRESSIVE MEDICINE, VOL. III., SEPTEMBER, 1901.—A Quarterly Digest of Advances, Discoveries and Improvements in the Medical and Surgical Sciences. Edited by Hobart Amory Hare, M.D., Professor of Therapautics and Materia Medica in the Jefferson Medical College of Philadelphia. Octavo, handsomely bound in cloth, 428 pages, 16 illustrations. Per annum, in four cloth-bound volumes, $10.00. Lea Brothers & Co., Philadelphia and New York.

This is a more than usually interesting volume. The most recent views on Pneumonia, Tuberculosis and other morbid conditions of the respiratory tract are set forth by Dr. Ewart. This chapter includes also a consideration of the disorders of the heart and blood-vessels, with an account of the newer methods of treatment.

Dr. Gottheil recounts the advances in Dermatology with an extended notice of the therapeutic use of the x-ray and light, under the captions radiotherapy and actinotherapy.

Diseases of the nervious system are taken up by Dr. Spiller, who devotes considerable space to tumors and abscesses of the brain.

Dr. Noble, under the department of obstetrics, gives recent views on eclampsia and symphyseotomy and the use of lumbar anesthesia.

SIMON'S MANUAL OF CHEMISTRY. — A guide to lectures and laboratory work for beginners in Chemistry, specially adapted for students of medicine, pharmacy and denistry. By W. Simon, Ph.D., M.D., Professor of Chemistry in the College of Physicians and Surgeons of Baltimore, in the Maryland College of Pharmacy, and in the Baltimore College of Dental Surgery. Seventh edition. Thoroughly revised and much enlarged. In one octavo volume of 613 pages with 66 engravings, one colored spectra plate and 8 colored plates representing 64 of the most important chemical reactions. Cloth, $3.00, *net.* Lea Brothers & Co., Publishers, Philadelphia and New York, 1901.

This popular work on chemistry has been brought to a seventh edition and has been revised and enlarged to meet the demands of advances in the science. The subjects of Chemical Physics and Physiological Chemistry have been presented more fully than in former editions. As an aid to laboratory work, a number of experiments have been added which may be performed by the students without much expense for chemical outfit.

---

### NEW SIGN OF MUMPS.

Tresilian mentions the fact that it is difficult in children to discover the opening of Stenson's duct, whereas in several cases of mumps under his observation it was plainly visible as a bright-red papilla.

